# Identification of conserved domains in the promoter regions of nitric oxide synthase 2: implications for the species-specific transcription and evolutionary differences

**DOI:** 10.1186/1471-2164-8-271

**Published:** 2007-08-08

**Authors:** Daniel Rico, Juan M Vaquerizas, Hernán Dopazo, Lisardo Boscá

**Affiliations:** 1 Centro Nacional de Investigaciones Cardiovasculares, Melchor Fernández Almagro 3, 28029 Madrid, Spain; 2 Bioinformatics Department, Centro de Investigación Príncipe Felipe, Autopista del Saler 16, 46013 Valencia, Spain; 3 Instituto de Investigaciones Biomédicas Alberto Sols (CSIC-UAM), Arturo Duperier 4, 28029 Madrid, Spain

## Abstract

**Background:**

The majority of the genes involved in the inflammatory response are highly conserved in mammals. These genes are not significantly expressed under normal conditions and are mainly regulated at the transcription and prost-transcriptional level. Transcription from the promoters of these genes is very dependent on NF-κB activation, which integrates the response to diverse extracellular stresses. However, in spite of the high conservation of the pattern of promoter regulation in κB-regulated genes, there is inter-species diversity in some genes. One example is nitric oxide synthase 2 (NOS-2), which exhibits a species-specific pattern of expression in response to infection or pro-inflammatory challenge.

**Results:**

We have conducted a comparative genomic analysis of NOS-2 with different bioinformatic approaches. This analysis shows that in the NOS-2 gene promoter the position and the evolutionary divergence of some conserved regions are different in rodents and non-rodent mammals, and in particular in primates. Two not previously described distal regions in rodents that are similar to the unique upstream region responsible of the NF-κB activation of NOS-2 in humans are fragmented and translocated to different locations in the rodent promoters. The rodent sequences moreover lack the functional κB sites and IFN-γ response sites present in the homologous human, rhesus monkey and chimpanzee regions. The absence of κB binding in these regions was confirmed by electrophoretic mobility shift assays.

**Conclusion:**

The data presented reveal divergence between rodents and other mammals in the location and functionality of conserved regions of the NOS-2 promoter containing NF-κB and IFN-γ response elements.

## Background

The biological activity of most genes involved in adaptive responses is regulated mainly at the level of transcription, and to a lower extent at the post-transcriptional level [1]. A primary example is the highly conserved mammalian inflammatory response, which involves the coordinated transcriptional induction of multiple genes. In this process, an important integrating role is played by the transcription factor NF-κB [2,3]. Extensive and detailed research has revealed common, evolutionarily conserved patterns in the regulation of NF-κB target genes [4-8]. However, the NOS-2 gene presents an exception. The NOS-2 coding region is highly conserved in all vertebrates [9,10], but its transcriptional regulation differs significantly, with a more restricted inducibility in primate species than that seen in rodents and other mammals. We have analyzed whether these different responses could be explained, at least in part, by divergent evolution of the NOS-2 promoter sequence.

Extensive studies of the mouse NOS-2 promoter have shown that only the proximal 1 kb sequence of the 5'-flanking region is necessary for complete inducibility by LPS and cytokine treatment [11-13]. To confer full promoter activity in the rat, 2 kb of additional 5' flanking region are required [14]. In contrast, the proximal region of the human NOS-2 promoter shows no inducibility: the proximal 3.7 kb sequence does not respond to LPS or cytokines in DLD-1 colon cells [15] or A549 lung epithelial cells [16]; and although the 4.7 kb upstream region has basal promoter activity in liver (AKN-1) and A549 cells, it does not show any cytokine-inducible activity [17]. These differences between human and rodent NOS-2 promoters correlate with differences in NOS-2 expression and NO synthesis, which is markedly less inducible in human cells.

Vera et al. (1996) [17] cloned 16 kb of the human NOS-2 5'-upstream flanking region and generated deletional NOS-2 promoter sequences ranging in size from 1.3 to 16 kb. Compared to the 1.3 kb sequence, they observed a 3-fold increase in the activity of promoter regions containing the -5.8 kb sequence, a 4-fold increase with the -7.2 kb sequence, and a 9-fold increase with the -16 kb sequence. Moreover, deletion of the region between -2.1 and -4.7 kb showed that this sequence lacks cytokine responsiveness.

NF-κB activation is required for cytokine induction of both human and rodent NOS-2. Mutational analysis of putative NF-κB sites in the 7.2 kb promoter region of the human NOS-2 promoter identified four κB sites between -5.2 and -6.1 kb, a region termed the distal NF-κB enhancer region [13,18]. We have compared the distribution of κB and other transcription factor binding sites (TFBSs) in the promoter region of NOS-2 in seven different mammals to evaluate their relative degree of evolutionary conservation and to investigate whether a pattern of changes in their promoter sequences could be established. For this analysis, we downloaded the corresponding promoter sequences from EnsEMBL. An 11 kb sequence spanning from -10 kb to +1 kb was first obtained from the Human Genome, and the available homologues in other species (orthologues) were then directly selected and downloaded. Using this strategy, we identified multiple conserved TFBSs that can be related to the activity of these promoters, at the time that we compared the evolutionary divergence in the enhancer and proximal region of the NOS-2 promoter to obtain information on the relative selective pressure on these sequences. Taken together, the data obtained are in agreement with the different inducibility of NOS-2 observed in mammals.

## Results

### Analysis of the promoter region of NOS-2 reveals different degrees of sequence conservation among mammals

**Figure 1:**
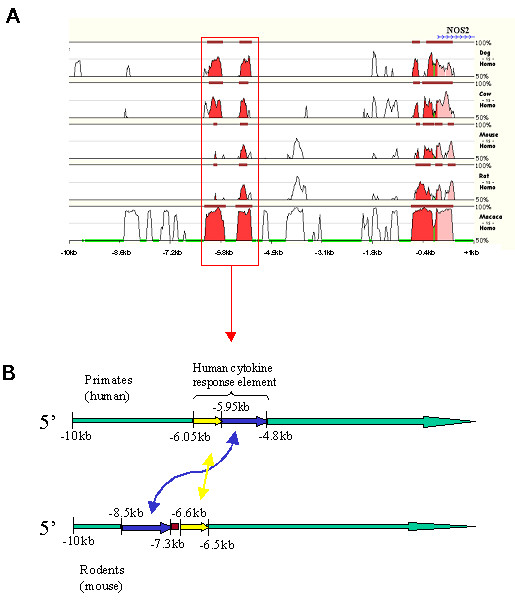
**(A) Standard stacked-pairwise visualization (smooth graph) of Mulan alignments of NOS-2 gene promoter**. The human sequence (from -10 kb to +1 kb) was selected as the reference species. Repeats were masked in all species with RepeatMasker (Mulan settings); green regions in the base sequence indicate the human repeats. The graphical representations of the other sequences are displayed according to their similarity to the base sequence: the closer they are to human, the higher is the conservation (top sequences are less conserved). Parameters selected for detection of evolutionarily conserved regions (ECR) were 90 bp minimum length and minimum similarity of 65% (50% bottom cut-off). Red indicates regions that are upstream from the transcription start site; pink regions are downstream from it. Two conserved motifs in rodent NOS-2 promoters indicate the presence of distal and fragmented sequences that are very similar to the unique enhancer region conferring NF-κB regulation in human NOS-2. (B) A schematic representation of the hypothetical translocation of these sequences in human and rodents; *double head arrows indicate the positional translocation*.

The -10 kb to +1 kb sequence of NOS-2 genes from different species were aligned by four independent methods to identify conserved regulatory sequences (see Methods). Mulan's graphical alignment is presented in Fig. 1A, with the human sequences as the reference. Although agreement was not exact, most Mulan aligned sequences coincided with the alignments generated by AVID and BlastZ (zPICTURE), and with MEME detected motifs. Because chimp is phylogenetically very close to human, most of the conserved regions were identical to their human counterparts. Consequently, analysis of these sequences was not very informative, and the alignments of chimpanzee sequences are not presented. In contrast, although human and murine gene promoters were generally similar, there were some significant differences. No similarities were found between any of the analyzed promoters in mammals and the available fish orthologues (tetraodon, fugu and zebrafish). The proximal region of the NOS-2 promoter was well conserved in all species analyzed, and two domains could be defined: one from -700 bp to +500 bp, the other from -1.3 kb to -1.0 kb. We also noted that whereas some regions were conserved only between dog and human (and therefore chimp), there was a region, located between -3 kb and -4 kb, that was conserved between rodents and primates but absent in dog. This analysis also showed that domains similar to the NF-κB enhancer region located between -5.2 and -6.1 kb in the human NOS-2 promoter [13,19] are also found in chimp, dog, cow, macaque, mouse and rat orthologues (Fig. 1A).

### The human NOS-2 distal NF-κB enhancer has two disrupted similar motifs in rodents

The MEME output revealed that the distal NF-κB enhancer region in the human NOS-2 promoter and the similar sequences in dog, mouse and rat contain two non-overlapping and very conserved motifs. Sequence analysis showed that this region is split in the rodent promoters (Fig. 1B) with one of the component motifs translocated further upstream (-7618 in mouse and -7388 in rat, compared to -5114 in human, -5552 in macaque, -6733 in cow and -4781 in dog). The position of the other motif was maintained positioning all the mammalian species examined (-6076 in human, -5981 in dog, -6524 in macaque, -7701 in cow, -6593 in mouse and -6649 in rat). When compared with human, dog and macaque, the whole region in cow was located further from the transcription start site (TSS). Nevertheless, it was not disrupted as in rodents. Mulan and zPICTURE also detected this fragmentation of the rodent sequence (Fig. 1A and data not shown), expanding their extension through the use of a gap-permitted alignment algorithm. AVID detected the conserved sequence of the -5 kb to -6.5 kb human NF-κB enhancer region in dog, cow and macaque, but did not detect the similar but fragmented sequences in rodents. This is probably due to the global alignment strategy used in AVID, which assumes that the order of the functionally conserved regions has been preserved [20].

### Evolutionary analysis the proximal promoter and enhancer regions reveals differences in rodents

**Figure 2:**
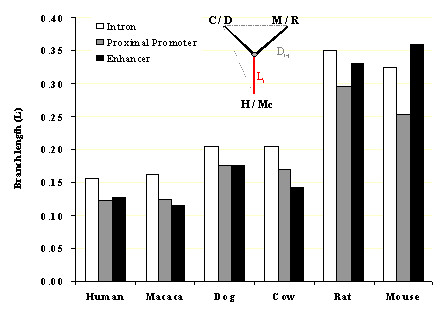
**Selective constraints deduced from distances in proximal promoter and enhancer**. Branch lengths derived from the evolutionary divergence of the proximal promoter (1 kb), the alignable enhancer and the fifth intron of the NOS-2 gene were compared for each one of the species. Two alternative and comparable evolutionary schemes using human-mouse-cow and macaque-rat-dog species were used. Branch length to species i (Li) was derived from pairwise distances (Di-j) according to an exact equation (see methods). See text for further explanation.

We calculated the evolutionary distance between every pair of species in the proximal promoter, in the enhancer region and in the fifth intron (the last for reference, as an approximation to the neutral rate of substitution). Afterwards, we computed the branch length for each sequences from the unique common ancestor for each trio of species using two phylogenetic schemes (see Methods). The results are shown in Figure 2, and they reveal that the proximal promoter and the enhancer have a similar branch length in primates and dog (cow presenting a branch even shorter in the enhancer). However, in mouse and rat the enhancer seems to evolve almost to the same rate than intron sequences. This result suggests that selective pressures are almost absent in the rodent enhancers, probably due to a loss of function in this region. Alternatively, the high efficiency of the rodent proximal promoter contributed to the loss of biological relevance of the enhancer regions (see Discussion).

### Bioinformatic analysis of κB sites and other inflammation-related TFBSs

The 11 kb NOS-2 promoter sequences were scanned with Match (TRANSFAC Professional 8.2) to ensure that the published κB sites were identified. Interestingly, high quality matrices from TRANSFAC (minimizing false positives) did not detect any κB sites in the human promoter, and only detected one in mouse (at -998 bp, described to be functional) and one in rat (at -2.46 kb), which has never been described in the literature. In view of these data, the promoters were scanned with all TRANSFAC vertebrate matrices. The results are summarized in Table 1. Four human elements were identified correctly; as Kleinert and co-workers pointed out [8] the functionality of the human κB at -119 is controversial. Three other functionally recognized human κB sites were not detected. In mouse and rat we found several sites, including the two functionally recognized murine κB sites. Strikingly, this analysis did not detect the described downstream and upstream κB sites in rat (highly similar to the mouse functional sites). Moreover, we found six putative sites in the rat promoter between -1601 and -2460. This is the region described by Zhang and co-workers to be important for full induction of NOS-2 by LPS and/or cytokines [14], although these authors' data indicated involvement of elements other than κB [21].

**Table 1:** NF-κB binding sites in NOS-2 promoters. Sequences were scanned with Match using TRANSFAC Professional 8.2 and minimizing false positives. Coincidence with experimentally demonstrated κB sites is indicated

	Position	Strand	Matrix match	Comments
Human	-119	+	0.908	Controversial (8)
	-4357	-	0.987	c-Rel.
	-5213	-	0.935	Described (13)
	-5802	-	0.908	Described (13)
	-8279	+	0.913	Described (19)
Mouse	+192	+	0.894	
	+191	+	0.913	
	-103	-	0.917	Described (11, 31)
	-996	+	0.989	c-Rel.
	-997	-	0.999	
	-998	+	0.979	Described (11, 31)
	-2796	+	0.918	
	-5347	+	0.971	
	-5343	-	0.967	
	-5342	+ & -	0.997	
Rat	-1601	-	0.917	
	-2394	-	0.989	c-Rel.
	-2399	+	0.993	
	-2458	+	0.989	c-Rel.
	-2459	-	0.999	
	-2460	+	0.979	
	-8999	+	0.980	c-Rel.
	-9000	-	0.937	
	-9655	-	0.936	

Since this approach still failed to detect previously recognized human κB sites, we defined a consensus κB sequence covering all functional κB elements described in NOS-2 promoters (NFKB-NOS2, see Methods). Scanning of NOS-2 promoters of human, dog, rat and mouse for this consensus sequence obviously detected all known human κB sites but the incidence of putative sites was extremely high: there are 79 sites that match that consensus sequence in human, 80 in chimpanzee, 71 in mouse, and 85 in rat (data not shown).

**Figure 3:**
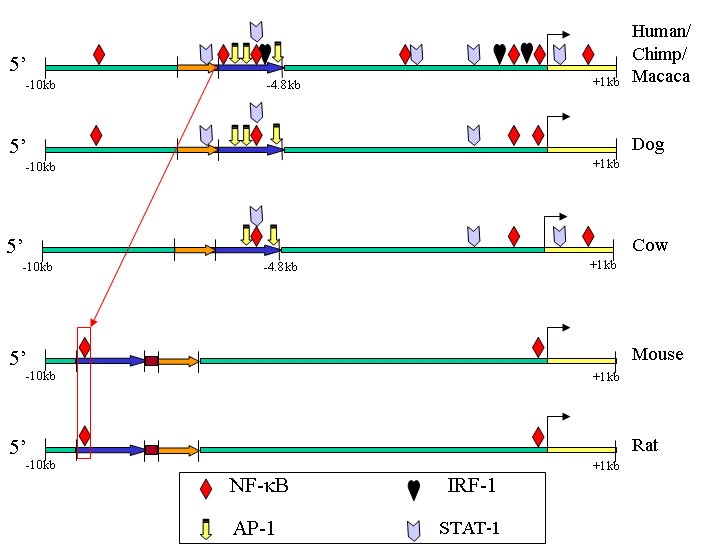
**Detailed analysis of NF-κB, AP-1 and IFN-γ dependent transcription factors**. Schematic representation of the relevant TFBSs in the promoter of NOS-2 from different species (plotted from additional data file 1). All the conserved sites in human and other mammals are shown, and conservation with other species is indicated.

NOS-2 is positively regulated by NF-κB, but also by IFN-γ dependent transcription factors (TFs) and AP-1, all of them key TFs for the regulation of the inflammatory response. In order to gain insight in the evolution of NOS-2 regulatory TFBSs, further analysis of NF-κB, AP-1 and IFN-γ regulated TFs was performed using MultiTF and TRANSFAC matrices. MultiTF is a phylogenetic footprinting tool interconnected with Mulan, a strategy that searches for TFBSs conserved among all the sequences aligned (i.e. multi-conserved sites). We included all mammal NOS-2 promoters in the analysis and tried all possible combinations of human vs. other mammal species alignments. A schematic representation of the results is shown (Fig. 3) and detailed information about these conserved TFBSs is available in additional file 1. It is remarkable the conservation of these sites in the distal regulatory domain in primates, dog and cow, but not in rodents. None of the human AP-1, IRF-1 and STAT-1 sites are conserved in the rodents' distal regions. Only the equivalent κB sites -5201 (human) and -8307 (rodents) are detected as conserved between primates and rodents with MultiTF, but we proved that this site is not functional in mouse by EMSAs (see below). A unique proximal conserved κB site was found in human that was present also in the dog and rodent NOS-2 promoters (at -115) and it corresponds to the functional κB binding sites from the promoters of mouse (at -110; -85 in the literature) and rat (at -71) NOS-2 genes. Sequence conservation of this site in human NOS-2 is interesting since apparently it is not functional. In this connection it might prove significant that the human and chimpanzee NOS-2 promoters contain an (A)n simple repeat downstream of this putative -115 κB site, between -94 and -75 bp. This sequence does not occur in dog, mouse or rat (alignments provided in the additional data file 2). This (A)n repeat in primates might be responsible, at least in part, for the lesser importance or loss of function of this human κB site (see Discussion).

### NF-κB does not bind to mouse motifs homologous to the human NF-κB enhancer region

**Figure 4:**
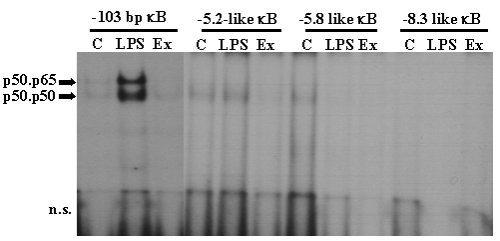
**EMSA of putative κB sites from the "human enhancer like" sequences of the mouse NOS-2 promoter**. The mouse -103 κB sequence was compared with the oligonucleotide sequences containing putative κB sites (identified as 'κB-like sites') in EMSAs with nuclear protein extracts from mouse peritoneal macrophages. Equal amounts of labeled oligonucleotide were used for each assay. C: nuclear extracts from control macrophages; LPS: nuclear extracts from macrophages treated with 200 ng/ml of LPS for 1 h. Ex: Extracts from LPS-treated cells in the presence of a 50-fold excess of unlabelled oligonucleotide. The bands corresponding to the p50/p65 and p50/p50 complexes are indicated. n.s. corresponds to the front and non specific binding in the EMSA.

All the previous data suggested that the distal rodent regions homologous to the human enhancer are probably not functional, at least not in response to pro-inflammatory stimuli. In contrast, the enhancer region is likely to be important not only in human, but also probably in other primates and dog too, based on their sequence similarity, position from the TSS and pattern of conserved TFBSs. As NOS-2 induction by LPS is totally dependent of NF-κB, we decided to test NF-κB binding to some regions in the mouse enhancer region by EMSA, in macrophages exposed to LPS. EMSA probes were designed based on mouse-human MEME alignments with the human sequences containing functional human κB sites. The functional mouse κB site at -103 (-87) was used as a positive control (Fig. 4). None of the three new putative mouse κB sequences tested was capable of binding NF-κB, and this probably indicates that this region does not operate as an NF-κB enhancer of NOS-2 transcription in mouse.

## Discussion

An immense amount of non-coding genomic sequence is now available from different species, allowing the comparison of large 5' upstream regions from orthologous genes. Functional promoter analysis is normally performed by cloning part of the 5' upstream region of the gene into an appropriate reporter vector, such as used for luciferase assays. The regions cloned are normally no longer than 2 or 3 kb, for two reasons. The first is that most regulatory elements are located close to the transcription start site. The second reason is technical: cloning of long inserts and cell transfection with large plasmids is complex.

The minimal promoter regions of many NF-κB regulated genes, as COX-2, IκBα or IκBβ, in mouse and human are located within the proximal 1 or 2 kb 5' upstream region, and the same applies to the mouse NOS-2 promoter. Thus for these genes, all the important regulatory regions are contained within this proximal 1 or 2 kb sequence. However, eukaryotic promoters frequently contain very distant enhancer elements that are important, or indeed sometimes essential, for the regulation of gene transcription [22]. This is the case with the human NOS-2 promoter, where maximal induction in luciferase assays requires a 16 kb promoter [17]. The effort of cloning this insert was necessary because human NOS-2 is poorly induced, or not induced at all, with shorter promoters. This is such a long sequence that the regulatory elements located between -9 kb and -16 kb still remain to be identified. Nevertheless, the important distal NF-κB enhancer region between -5.2 kb and -6 kb in the NOS-2 promoter, containing four κB sites, was described by Taylor and co-workers [13], and an additional κB site at -8293 bp was identified by Moss' group [19].

We were interested in exploring larger upstream regions of the NOS-2 promoter in mouse and other vertebrate species other than human, to see if we could find sequence similarities among them. Promoter length in eukaryotes is very variable; Nardone and co-workers [23] proposed that bioinformatic promoter analysis should extend, ideally, for 50–100 kb in either direction from the gene coding sequence, and should include introns and neighboring genes. Moreover, Dieterich and co-workers [24] estimated that 10 kb upstream from the transcription start site is sufficient in most cases. We selected 10 kb upstream plus 1 kb downstream from the TSS for a total of 11 kb per sequence, because this provides a good starting point with enough computational burdens. The 11 kb sequence used in this work contained all the described regulatory regions in human NOS-2 promoter, the longest known of all the promoters studied here. MEME (at the settings used) allowed us to detect 10 conserved motifs ranging from 6 to 120 base pairs, but did not give us information about the overall similarity between sequences; mVISTA, zPICTURE and Mulan provided this information. We preferred Mulan for graphical alignments and calculation of ECR lengths and similarities because it can detect sequence rearrangements (AVID/mVISTA cannot) and for its dynamic high power and flexibility. The Mulan graphical alignments are shown in Fig. 1A. MEME was very useful for highlighting the two motifs in rodents that were similar to the human distal NF-κB enhancer region, but which are separated, with one motif located further upstream, in the rodent sequences. This feature was also detected with Mulan and zPICTURE, and we present a schematic model in Fig. 1B to show the position of these regions in the human and mouse promoters. The human NF-κB enhancer region is entirely conserved in dog, cow and macaque; but unlike the case in rodents, there is one unique conserved region. The rodents are phylogenetically closer to human than to dog and cow [25], so we hypothesize that a translocation of part of this ancestral region has occurred in the rodent linage after its speciation from primates.

In addition to these strategies of analysis, we performed an evolutionary analysis in order to evaluate the selective pressures in the proximal promoter and the enhancer, to gain insight into functional differences among the species studied. The data obtained suggested a similar conservation of the enhancer and the proximal promoter in non-rodent species, while mouse and rat showed a comparative higher rate of evolution (in relation to intron sequences) suggesting lower selective constraint in these species.

These conserved distal sequences have not been previously described in rodents. MEME alignment with the κB sites from the human sequence identified three putative κB sites, but none showed any binding activity in EMSA (Fig. 3). The mouse κB-like probes differed from the homologous human sequences as follows: mouse -5.2 κB-like, 18 bp out of 28 bp equal to human (18/28); mouse -5.8 κB-like, 20/28; and mouse -8.3 κB-like, 13/26. Thus, these mouse sequences probably do not bind NF-κB because, despite the similarity of the regions where they are locate, the nucleotide substitutions in mouse sites are critical for NF-κB binding in EMSA. The effects of these sequence variations in the κB sites have been studied in depth and their affinity appears to be dependent on the nature of the co-activators engaged in the stabilization and activity of the NF-κB complexes [2].

These negative results are in agreement with the accepted idea that only the proximal 1000 bp of the 5'-flanking sequence is necessary for mouse NOS-2 promoter induction by NF-κB, while human NOS-2 requires the distal enhancer. The MultiTF results showed that not only is the overall structure of the human NF-κB enhancer region entirely conserved in dog, cow and primates, but also that some inflammatory TFBSs sites not present in rodents are conserved in the other species. We cannot be sure that mice lost these hypothetical ancestral κB sites, or whether the human κB sites were gained during evolution, but the analysis of multi-conserved TFBSs (Fig. 4) supports the first possibility. The functionality of these putative κB sites in macaque, dog and cow needs to be tested before making any conclusion, but if the sites do retain activity, that would support the hypothesis of a loss of functionality during the evolution of rodents.

Controversy exists about the functionality of the human proximal κB site located at -115 (additional file 1). Taylor and co-workers concluded that this site is non-functional, while others have reported a certain relevance (see [8]). We have found that this region is very conserved indeed (it corresponds to the multi-conserved κB site indicated after alignment of the human, dog and rodent NOS-2 promoters; additional file 2). Whether it is functional or not remains unclear, but it is certain that this region is less functional than the homologous site in mouse and rat. The (A)n simple repeat we found downstream of human and chimp putative -115 κB sites is absent in dog, mouse and rat (alignments in the additional files 2 and 3). The insertion of this element in primates may account for the reduced NF-κB activity of this site, as the insertion of transposable elements is an important evolutionary mechanism that can affect gene regulation [26]. Functional experiments and analysis of available sequences from more species will give us more data to test this hypothesis. In this regard, it should be mentioned that pathogens, as expected, have exerted a significant evolutionary pressure on NOS-2 inducibility, at least in mammalian macrophages. Although some controversy still persists [27-30], in vivo data demonstrate that non-rodent species display functional NOS-2 expression after infection with a wide array of pathogens. The relevant issue is the unusual deviation in the upstream region of rodent NOS-2 orthologues vs. other mammalian species. Solely evolutionary distances cannot explain this divergence since the same region is well-conserved in the more distant dog and horse orthologues. Availability of more NOS-2 upstream sequences from mammalian species might contribute to understand the evolutionary pressure exerted by pathogens.

MultiTF was a very powerful tool for this analysis because it can search common TFBSs in alignments of more than two sequences, and it allowed us to use TRANSFAC matrices and a consensus sequence defined by ourselves. However, bioinformatic discovery of putative TFBSs based on weight matrices (such as those in the TRANSFAC database) have serious drawbacks [31]. Attempts to minimize false positives and the use "high quality" matrices can fail to identify sites that have been experimentally demonstrated, as was the case with our analysis of the NOS-2 promoter. Indeed, phylogenetic footprinting of human-rodent alignments showed an acceptable degree of confidence [32] that is not the case for the NOS-2 promoter. Given these limitations, bioinformatic analysis of promoters needs to be complemented with functional data from wet-lab work. Even for very well known promoters, such as those studied in this paper, the functional data available for these types of studies is scarce. The results presented here represent a first basic approach to the study of vertebrate evolution of NF-κB regulated genes.

## Conclusion

The analysis of the promoter sequences of these inducible genes reveal differences in the degree of conservation, with NOS-2 showing divergence between rodents and other mammals in the location and functionality of conserved regions containing putative κB and IFN-γ elements. These data that are in agreement with the refractoriness in the induction of NOS-2 in primates and other non-rodent species.

## Methods

### Sequence data retrieval

Sequences of NOS-2 (NOS2A) were obtained from EnsEMBL [33]. The human gene sequence was downloaded from the Human Genome, and the NOS-2 orthologues in other species were directly selected from the Human GeneView page. In all cases, we examined the 10 kb region upstream from TSS at base +1 and the 1 kb downstream of this position. The EnsEMBL gene identities and chromosome positions sequence are listed in the additional data file 3. Sequences were obtained from EnsEMBL v22.1, except for dog sequences (v27.1 – Dec2004) and macaque and cow (v36 – Dec2005).

EnsEMBL gene discrepancies: The rat NOS-2 5'-UTR was not annotated in EnsEMBL, but we used the TSS described [34]. The start of transcription of mouse NOS-2 in EnsEMBL is 25 bp upstream with respect to the one first published [11,35]; it should be noted that although this site has been widely used in the literature, we used the TSS given for the EnsEMBL gene (ENSMUSG00000020826). Dog NOS-2 is only annotated in EnsEMBL as a fragment, and the exact transcription start site is uncertain (there are differences in the 5' end between NP_001003186 [GenBank:NP_001003186] and the genomic sequence in EnsEMBL (data not shown). We decided to position the dog NOS-2 +1 site further upstream than in the annotated gene in EnsEMBL. The same decision was made with macaque NOS-2. These decisions were based on the high genomic sequence similarity between dog (and macaque) sequence in this region and the start site of the human NOS-2 gene. The dog and macaque NOS-2 base positions are therefore only estimates; there are no experimental data on the dog or rhesus monkey NOS-2 promoter, and all results from these sequences presented in this work should be considered as a tentative approximation based on sequence comparison.

### Sequence alignment methods

We compared the different promoters with MEME [36], AVID at mVISTA [37-39], BlastZ with zPICTURE [40], and Mulan [41]. Mulan is based in the tba method [42]. We masked repetitive elements with RepeatMasker (A.F.A. Smit & P. Green, unpublished data, [43]) with the Mulan default settings, and we also used these masked sequences with all other methods. For clarity, we used the human sequence as the reference in AVID/mVISTA, zPICTURE and Mulan. The following settings were established in the analysis. MEME: one occurrence of a single motif per sequence among all sequences; motifs between 6 and 120 bp, 10 motifs to find. mVISTA: window length 90 bp, 65% minimum similarity. zPICTURE and Mulan: evolutionary conserved region (ECR) length 90 bp, 65% minimum similarity.

### Evolutionary analysis of the NOS-2 promoter

Calculation of the pairwise evolutionary distances between species was done for each one of the sequences (intron, enhancer an proximal promoter) by means of the Tajima-Nei method [44] using MEGA 3.1 program [45]. For each one of the sequences analysed, distances were transformed into branch lengths in a star-like unrooted tree by means of the following equation [46]: La = (Dab + Dac - Dbc)/2, where La is the length of the branch from the common ancestor leading to a and Dab, Dac, Dbc are the distances between species a and b, a and c, and b and c, respectively. Two alternative unrooted trees (trios of species) were considered: 1- human (H), mouse (M) and cow (C) and 2- macaque (Mc), rat (R) and dog (D). Since two of the species belongs to the same monophyletic group in both trees (primates and rodents), the internal node represents to the same common ancestor in both diagrams and consequently, branch lengths can be compared between sequences within species. It is important to emphasize that we are not considering that the phylogenetic relationships of these species is a star topology. We used this exact equation for determining the branch lengths of the three species, because the unique way to arrange three species in a phylogenetic tree is a star topology.

### TFBSs prediction

Match [47] was used, with false positives minimized with TRANSFAC Professional 8.2. MutiTF is integrated in Mulan [41], and uses an older version of TRANSFAC Professional 7.3. We used the default settings (optimized for function) with TRANSFAC matrices. NFKB-NOS2 consensus site was obtained from the alignment of all known functional κB sites in NOS-2 promoters of human, rat and mouse: GGRDNNNYYY.

### Protein extracts and EMSAs

Murine peritoneal macrophages were treated with 200 ng/ml of LPS for 1 h. Nuclear extracts and EMSAs were prepared as previously described [48]. The oligonucleotide sequences for binding were the following. Mouse -103 κB (-87 in [11,35]): sense, tcga TGC TAG GGG GAT TTT CCC TCT CTC TGT; antisense, tcga ACG ATC CCC CTA AAA GGG AGA GAG ACA. Mouse "-5,2 κB-like": sense, gatc CTG CCT GGG CAT GTC CAA CAA AAA CCC C; antisense gatc GGG GTT TTT GTT GGA CAT GCC CAG GCA G. Mouse "-5,8 κB-like": sense, gatc CTG GAG GCA GTT TCC AGA AGC ATG; antisense, gatc CAT GCT TCT GGA AAC TGC CTC CAG. Mouse "-8,3 κB-like": sense, gatc GAG GGC CAG TGG AGG GCC GGC AAG AG; antisense, gatc CTC TTG CCG GCC CTC CAC TGG CCC TC.

## Authors' contributions

DR participated in the design and carried out the sequence analysis and the experimental section. JMV and HD participated in the bioinformatic and statistical analysis. LB conceived of the study and participated in the design and coordination. All authors read and approved the final manuscript.

## Supplementary Material

Additional file 1 Supplement to Figure 3. The precise positions in human NOS-2 promoter of all the TFBSs shown in Fig. 3 Click here for file

Additional file 2 Proximal promoters of dog, mouse, rat and human. The alignment of the proximal promoters of dog, mouse, rat and human, showing the presence of a simple (A)n repeat next to the κB site in the human sequence. Click here for file

Additional file 3 EnsEMBL Gene IDs. All EnsEMBL Gene IDs and chromosomal locations of the sequences used in the study. Click here for file

## References

[B1] Kracht M, Saklatvala J (2002). Transcriptional and post-transcriptional control of gene expression in inflammation. Cytokine.

[B2] Natoli G (2006). Tuning up inflammation: how DNA sequence and chromatin organization control the induction of inflammatory genes by NF-κB. FEBS Lett.

[B3] Natoli G (2004). Little things that count in transcriptional regulation. Cell.

[B4] Ito CY, Kazantsev AG, Baldwin AS (1994). Three NF-k B sites in the  IκB-α promoter are required for induction of gene expression by TNF-α. Nucleic Acids Res.

[B5] Bates PW, Miyamoto S (2004). Expanded nuclear roles for IκBs. Sci STKE.

[B6] Algarte M, Kwon H, Genin P, Hiscott J (1999). Identification by in vivo genomic footprinting of a transcriptional switch containing NF-κB and Sp1 that regulates the IκBα promoter. Mol Cell Biol.

[B7] Tanabe T, Tohnai N (2002). Cyclooxygenase isozymes and their gene structures and expression. Prostaglandins Other Lipid Mediat.

[B8] Kleinert H, Schwarz PM, Forstermann U (2003). Regulation of the expression of inducible nitric oxide synthase. J Biol Chem.

[B9] Lin AW, Chang CC, McCormick CC (1996). Molecular cloning and expression of an avian macrophage nitric-oxide synthase cDNA and the analysis of the genomic 5'-flanking region. J Biol Chem.

[B10] Wang T, Ward M, Grabowski P, Secombes CJ (2001). Molecular cloning, gene organization and expression of rainbow trout (Oncorhynchus mykiss) inducible nitric oxide synthase (iNOS) gene. Biochem J.

[B11] Xie QW, Whisnant R, Nathan C (1993). Promoter of the mouse gene encoding calcium-independent nitric oxide synthase confers inducibility by interferon gamma and bacterial lipopolysaccharide. J Exp Med.

[B12] Lowenstein CJ, Alley EW, Raval P, Snowman AM, Snyder SH, Russell SW, Murphy WJ (1990). Macrophage nitric oxide synthase gene: two upstream regions mediate induction by interferon gamma and lipopolysaccharide. Proc Natl Acad Sci USA.

[B13] Taylor BS, de Vera ME, Ganster RW, Wang Q, Shapiro RA, Morris SM, Billiar TR, Geller DA (1998). Multiple NF-κB enhancer elements regulate cytokine induction of the human inducible nitric oxide synthase gene. J Biol Chem.

[B14] Zhang H, Chen X, Teng X, Snead C, Catravas JD (1998). Molecular cloning and analysis of the rat inducible nitric oxide synthase gene promoter in aortic smooth muscle cells. Biochem Pharmacol.

[B15] Laubach VE, Zhang CX, Russell SW, Murphy WJ, Sherman PA (1997). Analysis of expression and promoter function of the human inducible nitric oxide synthase gene in DLD-1 cells and monkey hepatocytes. Biochim Biophys Acta.

[B16] Marks-Konczalik J, Chu SC, Moss J (1998). Cytokine-mediated transcriptional induction of the human inducible nitric oxide synthase gene requires both activator protein 1 and nuclear factor κB-binding sites. J Biol Chem.

[B17] de Vera ME, Shapiro RA, Nussler AK, Mudgett JS, Simmons RL, Morris SM, Billiar TR, Geller DA (1996). Transcriptional regulation of human inducible nitric oxide synthase (NOS2) gene by cytokines: initial analysis of the human NOS2 promoter. Proc Natl Acad Sci USA.

[B18] Taylor BS, Geller DA (2000). Molecular regulation of the human inducible nitric oxide synthase (iNOS) gene. Shock.

[B19] Chu SC, Marks-Konczalik J, Wu HP, Banks TC, Moss J (1998). Analysis of the cytokine-stimulated human inducible nitric oxide synthase (iNOS) gene: characterization of differences between human and mouse iNOS promoters. Biochem Biophys Res Commun.

[B20] Ureta-Vidal A, Ettwiller L, Birney E (2003). Comparative genomics: genome-wide analysis in metazoan eukaryotes. Nat Rev Genet.

[B21] Zhang H, Teng X, Snead C, Catravas JD (2000). Non-NF-κB elements are required for full induction of the rat type II nitric oxide synthase in vascular smooth muscle cells. Br J Pharmacol.

[B22] Wray GA, Hahn MW, Abouheif E, Balhoff JP, Pizer M, Rockman MV, Romano LA (2003). The evolution of transcriptional regulation in eukaryotes. Mol Biol Evol.

[B23] Nardone J, Lee DU, Ansel KM, Rao A (2004). Bioinformatics for the 'bench biologist': how to find regulatory regions in genomic DNA. Nat Immunol.

[B24] Dieterich C, Cusack B, Wang H, Rateitschak K, Krause A, Vingron M (2002). Annotating regulatory DNA based on man-mouse genomic comparison. Bioinformatics.

[B25] Hedges SB (2002). The origin and evolution of model organisms. Nat Rev Genet.

[B26] Ackerman H, Udalova I, Hull J, Kwiatkowski D (2002). Evolution of a polymorphic regulatory element in interferon-gamma through transposition and mutation. Mol Biol Evol.

[B27] Fang FC, Nathan CF (2007). Man is not a mouse: reply. J Leukoc Biol.

[B28] Schneemann M, Schoeden G (2007). Macrophage biology and immunology: man is not a mouse. J Leukoc Biol.

[B29] Shibata T, Koide H, Hayashi R, Nagata K, Takeo C, Yoshida T, Noguchi Y, Tanaka T, Saito Y, Tatsuno I (2007). Molecular cloning and characterization of rat brain endothelial cell derived gene-1 (tumor suppressor candidate 5) expressing abundantly in adipose tissues. Mol Cell Endocrinol.

[B30] Weinberg JB (1998). Nitric oxide production and nitric oxide synthase type 2 expression by human mononuclear phagocytes: a review. Mol Med.

[B31] Wasserman WW, Sandelin A (2004). Applied bioinformatics for the identification of regulatory elements. Nat Rev Genet.

[B32] Sauer, Shelest E, Wingender E (2005). Evaluating phylogenetic footprinting for human-rodent comparisons. Bioinformatics.

[B33] (2007). EnsEMBL. http://www.EnsEMBL.org.

[B34] Keinanen R, Vartiainen N, Koistinaho J (1999). Molecular cloning and characterization of the rat inducible nitric oxide synthase (iNOS) gene. Gene.

[B35] Xie QW, Cho HJ, Calaycay J, Mumford RA, Swiderek KM, Lee TD, Ding A, Troso T, Nathan C (1992). Cloning and characterization of inducible nitric oxide synthase from mouse macrophages. Science.

[B36] Bailey TL, Elkan C (1994). Fitting a mixture model by expectation maximization to discover motifs in biopolymers. Proceedings of the Second International Conference on Intelligent Systems for Molecular Biology.

[B37] Bray N, Dubchak I, Pachter L (2003). AVID: A global alignment program. Genome Res.

[B38] Dubchak I, Brudno M, Loots GG, Pachter L, Mayor C, Rubin EM, Frazer KA (2000). Active conservation of noncoding sequences revealed by three-way species comparisons. Genome Res.

[B39] Mayor C, Brudno M, Schwartz JR, Poliakov A, Rubin EM, Frazer KA, Pachter LS, Dubchak I (2000). VISTA : visualizing global DNA sequence alignments of arbitrary length. Bioinformatics.

[B40] Ovcharenko I, Loots GG, Hardison RC, Miller W, Stubbs L (2004). zPicture: dynamic alignment and visualization tool for analyzing conservation profiles. Genome Res.

[B41] Ovcharenko I, Loots GG, Giardine BM, Hou M, Ma J, Hardison RC, Stubbs L, Miller W (2005). Mulan: multiple-sequence local alignment and visualization for studying function and evolution. Genome Res.

[B42] Blanchette M, Kent WJ, Riemer C, Elnitski L, Smit AF, Roskin KM, Baertsch R, Rosenbloom K, Clawson H, Green ED, Haussler D, Miller W (2004). Aligning multiple genomic sequences with the threaded blockset aligner. Genome Res.

[B43] Repeatmasker. http://www.repeatmasker.org.

[B44] Tajima F, Nei M (1984). Estimation of evolutionary distance between nucleotide sequences. Mol Biol Evol.

[B45] Kumar S, Tamura K, Nei M (2004). MEGA3: Integrated software for Molecular Evolutionary Genetics Analysis and sequence alignment. Brief Bioinform.

[B46] Dopazo H, Dopazo J (2005). Genome-scale evidence of the nematode-arthropod clade. Genome Biol.

[B47] Kel AE, Gossling E, Reuter I, Cheremushkin E, Kel-Margoulis OV, Wingender E (2003). MATCH: A tool for searching transcription factor binding sites in DNA sequences. Nucleic Acids Res.

[B48] Castrillo A, Traves PG, Martin-Sanz P, Parkinson S, Parker PJ, Bosca L (2003). Potentiation of protein kinase C ζ activity by 15-deoxy-Δ^12,14^-prostaglandin J_2_ induces an imbalance between mitogen-activated protein kinases and NF-κB that promotes apoptosis in macrophages. Mol Cell Biol.

