# Supplementary material for: Identification of conserved domains in the promoter regions of nitric oxide synthase 2: implications for the species-specific transcription and evolutionary differences
Source: BMC Genomics. 2007 Aug 8;8:271. doi: 10.1186/1471-2164-8-271 (PMC1973084; doi:10.1186/1471-2164-8-271)
Supplement: Additional file 1 — Supplement to Figure 3. The precise positions in human NOS-2 promoter of all the TFBSs shown in Fig. 3 [file 1471-2164-8-271-S1.doc]

Additional file 1

|  |  | Conserved TFBS present in | | | | |
| --- | --- | --- | --- | --- | --- | --- |
| TFBS | Human | Macaca | Dog | Cow | Mouse | Rat |
| NF-κB | +273 | Yes | - | Yes | - | - |
| STAT-x | +102 | Yes | - | Yes | - | - |
| NF-κB | -119 | Yes | Yes | - | Yes  -110 | Yes  -71 |
| IRF-1 | -396 | Yes | - | - | - | - |
| NF-κB | -464 | Yes | Yes | Yes | - | - |
| IRF-x | -556 | Yes | - | - | - | - |
| STAT-1 | -1536 | Yes | Yes | Yes | - | - |
| STAT-1 | -3647 | Yes | - | - | - | - |
| NF-κB | -3711 | Yes | - | - | - | - |
|  | -5106 | Yes | Yes | Yes | - | - |
| AP-1 | -5106 | Yes | Yes | Yes | - | - |
| STAT-1 | -5183 | Yes | - | - | - | - |
| IRF-1 | -5190 | Yes | - | - | - | - |
| STAT-x | -5206 | Yes | Yes | - | - | - |
| NF-κB | -5213 | Yes | Yes | - | - | - |
| AP-1 | -5292 | Yes | Yes | Yes | - | - |
| AP-1 | -5280 | Yes | Yes | - | - | - |
| NF-κB | -5801 | Yes | - | - | Yes  -8367 | Yes  -8125 |
| STAT-1 | -5947 | Yes | Yes | - | - | - |
| NF-κB | -8275 | Yes | - | - | - | - |

File format: PDF

Title: Detailed analysis of NF-κB, AP-1 and IFN-γ dependent transcription factors.

Description: The position of all the conserved sites in human and any of the other mammals are shown, and conservation with other species is indicated. A schematic representation is plotted is Fig. 4.
