# Supplementary material for: Identification of conserved domains in the promoter regions of nitric oxide synthase 2: implications for the species-specific transcription and evolutionary differences
Source: BMC Genomics. 2007 Aug 8;8:271. doi: 10.1186/1471-2164-8-271 (PMC1973084; doi:10.1186/1471-2164-8-271)
Supplement: Additional file 2 — Proximal promoters of dog, mouse, rat and human. The alignment of the proximal promoters of dog, mouse, rat and human, showing the presence of a simple (A)n repeat next to the κB site in the human sequence. [file 1471-2164-8-271-S2.doc]

Additional file 2

File format: PDF

Title: Mulan multiple alignment of NOS-2 promoters (proximal region)

Description: Presence of (A)n simple repeat only present in primates; the repeat is also present in chimp (data not shown).

**A. Repeats masked:**

Dog CACAGAGTGATGTAACAACAAGGTCAGGTCACCCATAGGCCAGGAGCCCCAGGCCAGAAG

Mouse CACAGAGTGATGTAATCA-----------AGCACACAGA--------------CTAGGAG

Rat CACAGAGTGACGTAATAA-----------TGCATACAGA--------------CTAGGAG

Human CACAGAGTGATGTAACAGCAAGATCAGGTCACCCACAGGCC------------CTGGCAG -151

********** **** * * ** * * **

κB site

Dog TCCCAGTCATAAATTAGCAAAC-TGTACACCCAGCCCGGGACACTCCCTTTGGAAA----

Mouse TGTCCATCATGAATGAGCTAACTTGCACACCCAACTGGGGACTCTCCCTTTGGGAA----

Rat TGTCCATCGCGAATGAGCTAACTTGCACACCCTACTGGGGACTCTCCCTTTGGGAA----

Human TCACAGTCATAAATTAGCTAAC-TGTACA-CAAGCTGGGGACACTCCCTTTGGAAACCNN -93

* * ** *** *** *** ** *** * * ***** ********** **

Dog ----------AATAAAAAGAGACCTTTATGCAAAAACGGCTCCCTGGAATGATGTGGGGT

Mouse ----------------CAGT------TATGC-AAAATAGCTCTGCAG---AGCCTGGAG-

Rat ----------------CAGTGA-CTTTATGC-AAAACAGCTCTGCAG---AGCGTGGAT-

Human NNNNNNNNNNNNNNNNNNGAGACCTTTATGCAAAAACAACTCTCTGG-ATGGCATGGGGT -34

* ***** **** *** * ***

**B. No masked sequences:**

Rat AA-----------TGCATACAGA--------------CTAGGAGTGTCCATCGCGAATGA

Dog AACAAGGTCAGGTCACCCATAGGCCAGGAGCCCCAGGCCAGAAGTCCCAGTCATAAATTA

Mouse CA-----------AGCACACAGA--------------CTAGGAGTGTCCATCATGAATGA

Human AGCAAGATCAGGTCACCCACAGGCC------------CTGGCAGTCACAGTCATAAATTA -135

* * ** * * *** * ** *** *

κB site

Rat GCTAACTTGCACACCCTACTGGGGACTCTCCCTTTGG--------------------GAA

Dog GCAAAC-TGTACACCCAGCCCGGGACACTCCCTTTGG--------------AAAAATAAA

Mouse GCTAACTTGCACACCCAACTGGGGACTCTCCCTTTGG--------------------GAA

Human GCTAAC-TGTACA-CAAGCTGGGGACACTCCCTTTGGAAACCAAAAAAAAAAAAAAAAAA -77

** *** ** *** * * ***** ********** **

Rat CAGTGA-CTTTATGC-AAAACAGCTCTGCAG---AGCGTGGAT-GGGTATAAATACCTG-

Dog AAGAGACCTTTATGCAAAAACGGCTCCCTGGAATGATGTGGGGTGGGTATAAATACCTCT

Mouse CAGT------TATGC-AAAATAGCTCTGCAG---AGCCTGGAG-GGGTATAAATACCTG-

Human AAGAGACCTTTATGCAAAAACAACTCTCTGG-ATGGCATGGGGTGAGTATAAATACTTC- -19

** ***** **** *** * *** * ********** *
