# Supplementary material for: Identification of conserved domains in the promoter regions of nitric oxide synthase 2: implications for the species-specific transcription and evolutionary differences
Source: BMC Genomics. 2007 Aug 8;8:271. doi: 10.1186/1471-2164-8-271 (PMC1973084; doi:10.1186/1471-2164-8-271)
Supplement: Additional file 3 — EnsEMBL Gene IDs. All EnsEMBL Gene IDs and chromosomal locations of the sequences used in the study. [file 1471-2164-8-271-S3.doc]

## Additional file 3

## File format: PDF

## Title: EnsEMBL Gene ID’s and chromosomal locations of the sequences used in the study.

## Description: data localizing NOS2 gene in several animal species.

| **Gene** | **Species** | **Ensembl Gene ID** | **Position** |
| --- | --- | --- | --- |
| NOS2A | Chicken | ENSGALG00000005693 | 19.8749269-8759268 |
| NOS2A | Chimp | ENSPTRG00000008902 | 19_random.12380530-12390529 |
| NOS2A | Dog | ENSCAFG00000018642 | 9.34809324-34820323 |
| NOS2A | Homo | ENSG00000007171 | 17.23107922-23151682 |
| NOS2A | Mouse | ENSMUSG00000020826 | 11.78482117-78492116 |
| NOS2A | Rat | ENSRNOG00000011023 | 10.65040474-65051473 |
| NOS2A | Zebrafish | ENSDARG00000005889 | 4.3028648-3038647 |
| NOS2A | Macaque | ENSMMUG00000010083 | scaffold_67.1033-51843 |
| NOS2A | Cow | ENSBTAG00000006894 | 19.12857695-12868695 |
